# Study on Flexural Fatigue Properties of POM Fiber Airport Pavement Concrete

**DOI:** 10.3390/polym14152979

**Published:** 2022-07-22

**Authors:** Zhenhui Wang, Rongxin Guo, Guoshou Liu, Luxin Guo, Yong Yan

**Affiliations:** 1 Faculty of Civil Engineering and Mechanics, Kunming University of Science and Technology, Kunming 650504, China; 18313304610@163.com (Z.W.); guorx@kmust.edu.cn (R.G.); liugshfirst@126.com (G.L.); guoluxin2019@163.com (L.G.); 2 Yunnan Key Laboratory of Disaster Reduction in Civil Engineering, Kunming 650504, China

**Keywords:** airport pavement concrete, polyoxymethylene fiber, flexural fatigue, Weibull distribution function, life prediction equation

## Abstract

Polyoxymethylene (POM) fiber is a new polymer fiber with the potential to improve the performance of airport pavement concrete. The effect of POM fiber on the flexural fatigue properties of concrete is an important issue in its application for airport pavement concrete. In this study, four-point flexural fatigue experiments were conducted using ordinary performance concrete (OPC) and POM fiber airport pavement concrete (PFAPC) with fiber volume contents of 0.6% and 1.2%, at four stress levels, to examine the flexural fatigue characteristics of these materials. A two-parameter Weibull distribution test of flexural fatigue life was performed, after examining the change in flexural fatigue deformation using the cycle ratio (*n*/*N*). A flexural fatigue life equation was then constructed considering various failure probabilities (survival rate). The results show that POM fiber had no discernible impact on the static load strength of airport pavement concrete, and the difference between PFAPC and OPC in terms of static load strength was less than 5%. POM fiber can substantially increase the flexural fatigue deformation capacity of airport pavement concrete by almost 100%, but POM fiber had a different degree of detrimental impact on the fatigue life of airport pavement concrete compared to OPC, with a maximum decrease of 85%. The fatigue lives of OPC and PFAPC adhered to the two-parameter Weibull distribution, the single- and double-log fatigue equations considering various failure probabilities had a high fitting degree based on the two-parameter Weibull distribution, and their *R*^2^ was essentially over 0.90. The ultimate fatigue strength of PFAPC was roughly 4% lower than that of OPC. This study on the flexural fatigue properties of POM fiber airport pavement concrete has apparent research value for the extension of POM fiber to the construction of long-life airport pavements.

## 1. Introduction

Cement concrete is often used for airport pavement structures. Presently, aircraft are carrying increasingly heavy loads, and takeoffs and landings happen more frequently, so the load on airport pavement structures is becoming increasingly severe; cracking, angle loss, and plate fractures frequently occur during the course of operations [1]. As such, the performance of airport pavement concrete must be improved. During use, airport pavement concrete is subject to impact load as well as aircraft fatigue load. Concrete deterioration under fatigue stress is one of the primary causes of structural failure and a main contributor to structural durability damage [2].

Based on a trabecular three-point flexural test, Zheng et al. [3] investigated the flexural fatigue of high-strength steel–fiber polymer concrete, and discovered that the addition of 0.64 wt% steel fiber and 0.015 wt% polymer latex substantially increased concrete toughness and flexural fatigue resistance. However, few test results were available at that time. For design purposes, the stress-level–fatigue-life (*S*–*N*) curve could not be created. On the basis of the four-point flexural testing of trabecular beams, Lv [4] investigated the flexural fatigue characteristics of glass-fiber concrete, and discovered that as the glass-fiber content increased, so did the ability of the concrete to withstand flexural fatigue. The fatigue life of glass-fiber concrete adhered to the two- and three-parameter Weibull distributions for each fiber content and stress level, and the matching *S–N* curve was established. According to Liu et al. [5], the bilinear fatigue properties of polyvinyl alcohol (PVA)-fiber-reinforced ultra-high-toughness cement-based composites (UHTCC) are comparable to those of metal materials, despite the flexural fatigue life of these materials obeying a two-parameter Weibull distribution. The fatigue life of UHPC increases with the increase in steel fiber content at the same stress level, according to Niu [6], who discovered this using a four-point flexural fatigue test with a trabecular beam. Additionally, the fatigue life of UHPC obeys a two-parameter Weibull distribution. The fatigue life of concrete modified with polyester (FC) fiber and PVA fiber increased 1–2 times, and the fatigue life of fiber airport pavement concrete obeys a two-parameter Weibull distribution, according to Li [7], who investigated the effects of FC, PVA, and polypropylene (PP) fibers on the flexural fatigue performance of airport pavement concrete. He [8] studied the effect of basalt fiber length and content on the three- and four-point flexural fatigue properties of concrete under various stress levels, using a finite element simulation. According to the calculated results, fiber content had the strongest impact on concrete fatigue. The results of calculations for three- and four-point flexural fatigue were largely in agreement. According to Rios et al. [9], a high steel-fiber content decreased overall matrix porosity and pore size, which shortened UHPC fatigue life and increased fatigue strength by roughly 78%. Although hybrid PP fiber increases fatigue life dispersion, it has little impact on fatigue strength. According to Yang et al. [10], basalt fiber can enhance concrete’s fatigue properties under high levels of stress fatigue load by altering the direction of internal fracture development and lengthening the crack propagation path.

Steel fiber, basalt fiber, glass fiber, and other synthetic fiber concretes have all been studied to reveal their fatigue properties. Polyoxymethylene (POM) is a synthetic fiber with strong mechanical strength, dimensional stability, strong alkali resistance, and chemical corrosion resistance [11,12]. Table 1 displays the characteristics of POM fiber and other widely used fibers [11,13,14,15]. POM fiber is more hydrophobic than natural fiber [16], which helps to maintain the working performance of freshly mixed concrete. In comparison with other fibers, research into POM fiber in cement-based composites started later; however, owing to its superior mechanical and dispersion properties, POM fiber has rapidly become a research hotspot. Through a series of tests on POM-fiber-reinforced materials, numerous researchers have investigated the impact of the inclusion of POM fiber on various properties of cement-based composites, including mechanical properties [17], durability [18,19], and high-temperature performance tests [20,21]. An essential technical indicator of the status of POM fiber airport pavement concrete (PFAPC) is its resistance to fatigue. For airport pavements to safely operate over a long lifespan, the fatigue properties of this new material under flexural load must be assessed.

In this study, PFAPC trabeculae with two different POM fiber contents, along with ordinary performance concrete (OPC) trabeculae, were subjected to four-point flexural fatigue testing. Under various load levels, the flexural fatigue deformation and fatigue life were measured. The fatigue life of these materials was statistically investigated, the relevant fatigue equation was created, and the flexural fatigue deformation properties were examined under various cycles. Then, the final fatigue strength was calculated.

## 2. Materials and Methods

### 2.1. Raw Materials and Mix Proportion

**Figure 1:**
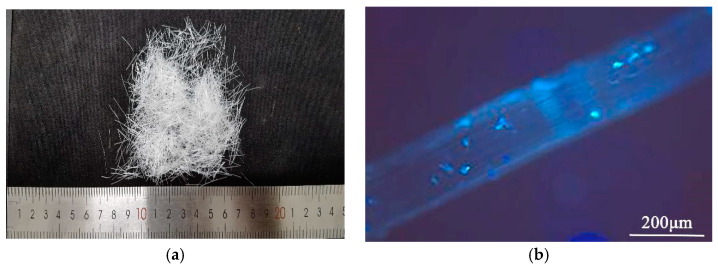
(**a**) Macro and (**b**) micro morphologies of POM fibers.

The mix ratio of OPC was calculated in accordance with the specifications for airport cement concrete pavement design (MH/T 5004-2010) [22]. To increase the workability and early performance of a dry and rigid concrete mixture for airport pavement concrete, P·O 42.5 cement, 4.75~16 and 16~26.5 mm double-graded gravel, and machine-made sand with a fineness modulus of 3.1 were used. F-type I low-calcium fly ash, S95-grade blast furnace slag powder, and polycarboxylic acid high performance water reducing agent were also added (fly ash replaced 16% of the cement mass, slag powder replaced 11% of the cement mass, and water reducing agent comprised 0.5% of the binding material’s total mass). The POM fiber was obtained from Chongqing Yuntianhua Tiamjuxincai Co., Ltd. (Chongqing, China); Table 2 displays its physical and mechanical properties. Figure 1 depicts the shape of POM fiber; the microscopic images were obtained using a ZEISS microscope (Carl Zeiss AG, Oberkochen, Baden-Württemberg, Germany). POM fibers were introduced to the fresh concrete at 0.6 and 1.2% of the OPC volume. After being thoroughly agitated, the fresh concrete was packed into a 100 × 100 × 400 mm beam for vibration compaction and mold testing. The four-point flexural fatigue test was performed 24 h after the product had been poured. The product was withdrawn from the mold and placed at a temperature range of 20 ± 2 °C where the relative humidity was above 95%, for standard curing over a period of 90 days. Additionally, a 100 × 100 × 100 mm cube and a beam with the same size as the fatigue specimen were created, to test the compressive and flexural strengths at 28 and 90 days. Table 3 displays the mix proportion of the specimens.

### 2.2. Test Method

Before the test, the specimen surface was polished using a hand-held concrete grinder after being removed from the curing room. A high-pressure pneumatic sprayer was used to spray a thin layer of epoxy resin evenly on the surface of specimens, to reduce the test error produced by the unevenness of the surface. The static load strength was established prior to the fatigue test. The flexural and fatigue tests were performed using an MTS 810 test machine (MTS Systems Corporation, Eden Prairie, MN, USA), and the compression test using hydraulic pressure testing equipment with a loading rate of 18 kN/s. To produce roughly one-dimensional stress conditions in the center of the specimen, the purely flexural part of the four-point loading method was used. The specimen was loaded at a rate of 1 mm/min until damaged was observed, with loading points spaced 100 mm apart. To choose the upper and lower limits of loading that corresponded to the stress levels in the fatigue test, the failure strength was determined by taking the average value of three recordings.

**Figure 2:**
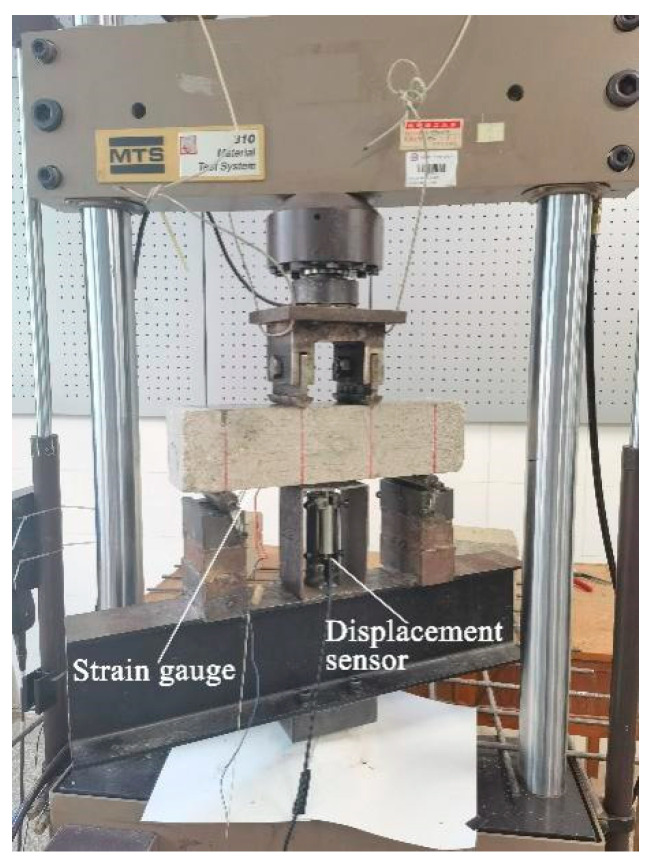
Loading setup photograph.

**Figure 3:**
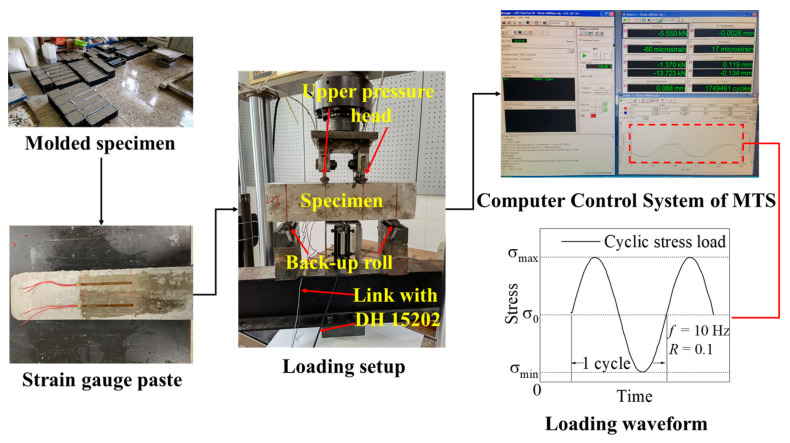
Experimental flowchart.

In this study, repeated loading at a frequency of 10 Hz was used, corresponding to a commercial airliner taxiing at a speed of 20 km/h on a runway [23], with constant amplitude sine wave loading during the fatigue test loading to simulate the actual waveform of airport surface stress [24]. The cyclic characteristic value (*R*) of fatigue load for concrete roads, bridges, and other structures is 0.1 (the minimum fatigue loading stress is 10% of the maximum stress) [25]. The stress scale (*S*) was 0.65, 0.70, 0.75, and 0.80. Additionally, two strain gauges and a displacement sensor were placed at the bottom of each specimen, along the symmetry axis of the pure bending section. A dynamic strain tester (DH 15202, Donghua Testing Technology Co., Ltd., Jingjiang, Jiangsu, China) was attached to the test instrument. The recorded loading load, tensile strain in the pure bending region at the bottom of the beam prior to breaking, and midspan deflection deformation were simultaneously recorded during the loading procedure. To ensure that the epoxy glue on the contact surface was compacted and in complete contact with the loading point of the testing apparatus, preloading (10 kN) was repeatedly performed before the fatigue test began. Figure 2 displays a photograph of the loading setup. Figure 3 displays an experimental flowchart. Five specimens were chosen for each loading condition in the fatigue test, and the data were deleted and supplied with significant deviations to confirm the validity of the data on the fatigue life of the five specimens, as previously suggested [26].

## 3. Results and Discussion

### 3.1. Static Test Result

Table 4 displays the static characteristics of each group of concrete specimens at the test ages.

Table 4 shows that the compressive strength of PFAPC with 0.6 and 1.2% POM fibers in comparison with OPC ranged from −9.7 to −8.2% at 28 days, and 8.9 to 10.2% at 90 days, respectively. The flexural strength at 28 days was between −3 and 1.3%, whereas the flexural strength at 90 days was between −2 and 2.1%. The POM fiber had no discernible impact on the ability of the concrete airport pavement to withstand static loads.

### 3.2. Flexural Fatigue Deformation

**Figure 4:**
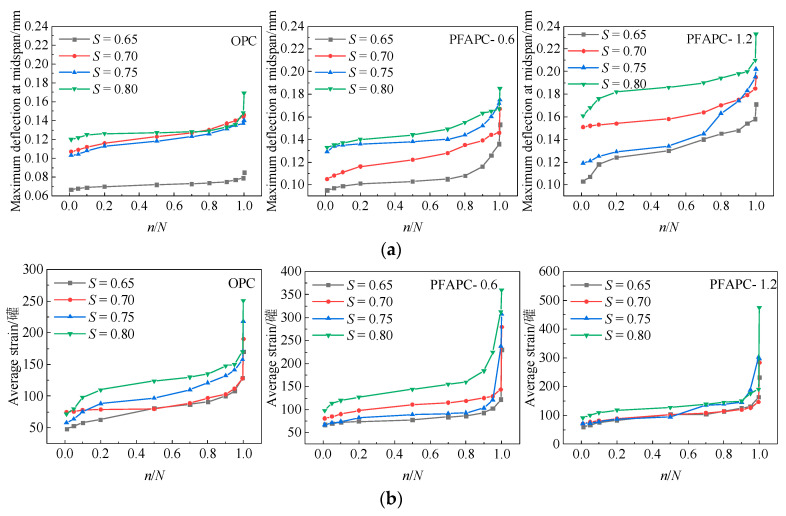
Curves of specimen deformation with cyclic ratio: (**a**) maximum deflection at midspan; (**b**) average strain.

In this study, the average bottom tensile strain and maximum midspan deflection of specimens were employed to describe the fatigue deformation at various stress levels. In Figure 4, the maximum midspan deflection and average tensile strain at the bottom are shown on the ordinate; the variation curves of specimen deformation were generated on the abscissa with the cyclic ratio *n*/*N* (the ratio of current cycle times to fatigue life) based on 11 data points. The maximum midspan deflection and average tensile strain at 1, 5, 10, 20, 50, 70, 80, 90, 95, 99.5 and 100% of specimen fatigue life at each stress level represent the 11 selected data points [27].

The maximum midspan deflection and average tensile strain of OPC and PFAPC both increased as the cycle ratio increased, and their deformation characteristics were broadly categorized into three stages [4,5,27,28]: development, stable, and failure stages, as shown in Figure 4. The interior microcracks of the material started to increase under fatigue stress during the development stage (often before a 20% cyclic ratio). The maximum midspan deflection and average tensile strain rapidly increased with increasing cycle ratio, as a result of the cracks in the weak region of the matrix. Fewer fissures were present at this point, and the material began to suffer interior damage. The maximum midspan deflection and average tensile strain gradually and consistently increased, and new fractures started to emerge in the material during the stable period (generally at a cycle ratio between 20% and 80%). The damage to the material expanded at a stable rate. In the failure stage (mainly at a cycle ratio greater than 80%), sharp increases were observed in specimen instability, internal damage across the maximum deflection, and the average tensile strain. The stress level and the average tensile strain specimen failure (when the average tensile strain was 100%) increased with increasing POM fiber content, increasing average maximum deflection, and increasing failure in specimen cross tensile strain. In this phase, the main crack matrix was internal, but due to the influence of POM fiber bridge cracks, the front of the specimen could absorb more destructive energy, delaying specimen destruction. According to Liu et al. [5], the main crack width matrix is responsible for the stage of accelerated fatigue deformation. PFAPC is tougher under fatigue load than OPC. At the 0.80 stress level, the maximum midspan deflection and average tensile strain of the 0.6% PFAPC group increased by 9.5% and 43.4%, respectively; those of the 1.2% PFAPC group increased by 37.9% and 89.2%, respectively. Under the 0.65 stress level the maximum midspan deflection and average tensile strain of the 0.6% dosage group increased by 80% and 35.3%, respectively, whereas those of the 1.2% dosage group increased by 101.2% and 36.5%, respectively. The average tensile strain growth rate of OPC and PFAPC accelerated as the stress level increased, particularly in the failure stage, as shown in Figure 4b. Additionally, as evidenced by fatigue deformation test findings for the majority of fiber-reinforced concrete materials [4,5,27], differing stress levels have no appreciable impact on the fatigue deformation of specimens within a group.

### 3.3. Fatigue Life Probability Distribution

Weibull distribution was used for assessing the distribution of the fatigue life of concrete materials [29]. The two-parameter Weibull distribution equation can be condensed into Equation (1) [30,31]:

(1) $$\ln\left[\ln\left(1/p\right)\right]=b\ln N-b\ln N_{a}$$

because Y=ln[ln(1/p)], X=lnN, $\beta =b\ln N_{a}$. Then, Equation (1) can be written as:

(2) Y=bX−β

where *b* is the Weibull shape parameter (slope parameter), *p* is the reliability, *N* is the fatigue life, and *N_a_* is the characteristic life parameter. As Equation (2) is a linear equation, each Weibull parameter can be directly determined from the fitting line. Two-parameter Weibull distribution of experimental data is considered valid if regression analysis demonstrates a strong linear relationship between *Y* and *X*. Table 5 displays the fatigue life for each stress level, where the data for fatigue life (*n*) from each stress level are organized from small to large, starting with serial number *i*. Equation (3) can be used to compute the reliability *p* related to the fatigue life *N*:

(3) $$p=1-\frac{i}{n+1}$$

**Figure 5:**
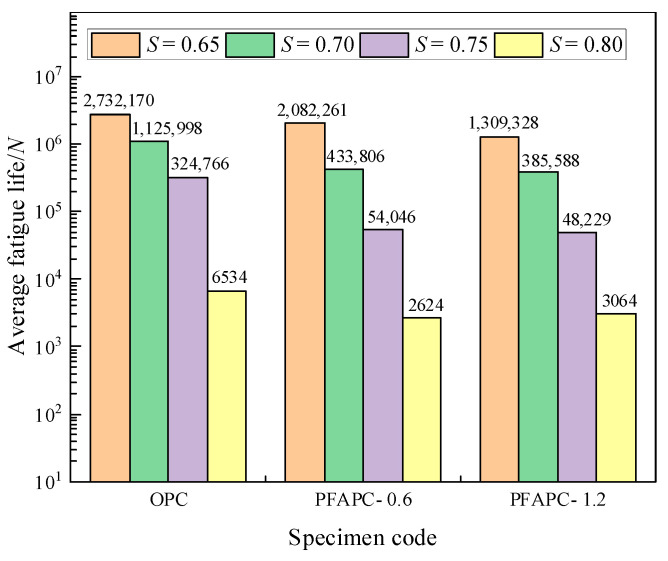
Average fatigue life.

The averaged fatigue life values from Table 5 are depicted as a histogram in Figure 5.

Figure 5 shows that when the stress level was 0.65, the fatigue life of concrete with POM fibers at 0.6 and 1.2% volume reduced by 23.8 and 52.1%, respectively; when the stress level was 0.70, the fatigue life reduced by 61.5 and 65.8%, respectively; when the stress level was 0.75, the fatigue life reduced by 83.4 and 85.1%, respectively; and when the stress level was 0.80, the fatigue life reduced by 59.8 and 53.1%, respectively. The concrete fatigue life of airport pavement considerably decreased after POM fiber was introduced. The fatigue life of the 1.2% dosage group was shorter than that of the 0.6% dosage group, except at a stress level of 0.80. Contrary to the findings of most fiber concrete fatigue tests, POM fiber did not extend the fatigue life of airport pavement concrete: the inclusion of POM fiber substantially reduced the fatigue life. No authoritative research findings have yet been published on the fatigue performance of POM fiber concrete. Both types of POM fibers generally hadshorter fatigue lives than regular concrete. POM fibers did not increase the fatigue life of airport pavement concrete according to the parameter values used in this investigation.

**Figure 6:**
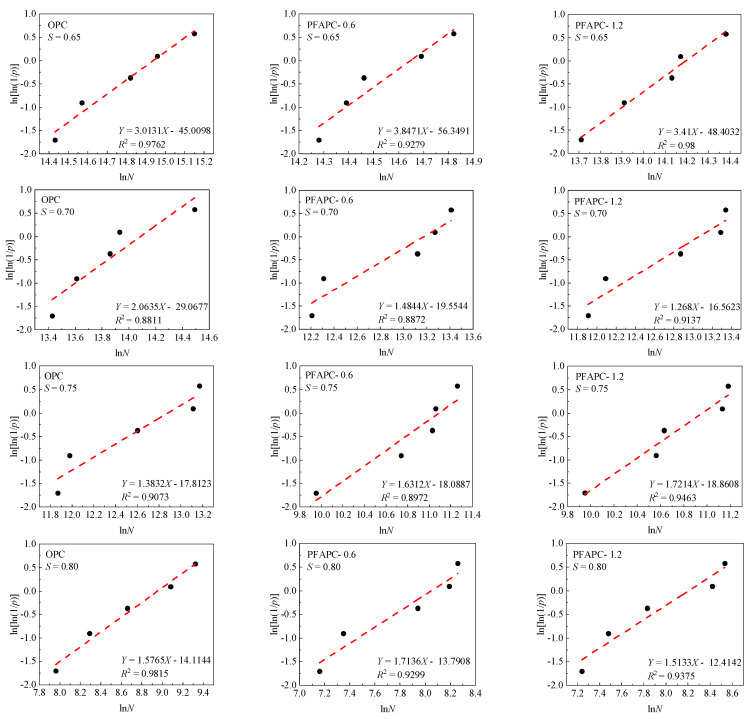
Two-parameter Weibull distribution of fatigue life test results.

According to the information in Table 5, the ln[ln(1/*p*)]-ln*N* curves of OPC and PFAPC were developed with varying fiber contents under various stress levels, as shown in Figure 6.

Figure 6 shows that the OPC and PFAPC test data fitting lines were linear at different stress levels. A strong, statistically significant linear relationship was found between ln[ln(1/*p*)] and ln*N*, and the correlation coefficient *R*^2^ was greater than 0.9. The fatigue life could be described by a two-parameter Weibull distribution. Table 6 provides a summary of the Weibull distribution parameters of OPC and PFAPC at various stress levels, as shown in Figure 6.

### 3.4. Test of Fitting Degree of Fatigue Life Probability Distribution

The Kolmogorov–Smirnov (K–S) method was used to test the degree of fatigue life fit, to confirm the utility of the two-parameter Weibull distribution for assessing the fatigue life distribution of OPC and PFAPC. Small sample sizes (less than 20) are better-suited for the K–S test [32]. Equation (4) expresses the K–S technique:

(4) $$D_{i}=\underset{i=1,\cdot \cdot \cdot ,k}{\max}\left[\left|F^{*}\left(x_{i}\right)-P_{f}\left(N_{i}\right)\right|\right]$$

(5) $$\mathrm{there}\;\mathrm{into},\;F^{*}\left(N_{i}\right)=i/n$$

(6) $$P_{f}\left(N_{i}\right)=1-\exp\left[-{\left(\frac{N_{i}}{N_{a}}\right)}^{b}\right]$$

where *N_i_* is the fatigue life associated with serial number *i*; the coefficient *b* and the life characteristic *N_a_* were substituted into the preceding formula. Table 7 summarizes the results of the fitting test of the two-parameter Weibull distribution of OPC and PFAPC.

By checking the K–S critical value table, when the sample size *n* was five and the significance level was 0.05, the critical value *D_c_* was 0.563, the observed statistical values *D_i_* of each group were all less than *D_c_*, and the K–S test results was accepted, which further verified that the fatigue life distribution of OPC and PFAPC followed the two-parameter Weibull distribution. The significance level was less than 5%.

### 3.5. Flexural Fatigue Equation

The single-logarithm (*S*-lg*N*) and double-logarithm (lg*S*-lg*N*) fatigue equations for concrete are frequently used by engineers to match fatigue performance curves. The single-logarithm fatigue equation is as follows [33]:

(7) S=A−BlgN

The fatigue equation has two boundary conditions [33]:

(8) $$\begin{array}{l} S=1\left(N=1\right)\\ S\to 0\left(N\to \infty \right) \end{array}$$

The second boundary condition of the single-log fatigue equation cannot be satisfied, preventing the determination of the fatigue properties of concrete at low stress levels (*S* < 0.50). The double-log fatigue equation [30] is established to prolong the fatigue curve in the direction of *S*→0:

(9) lgS=lga−blgN

**Figure 7:**
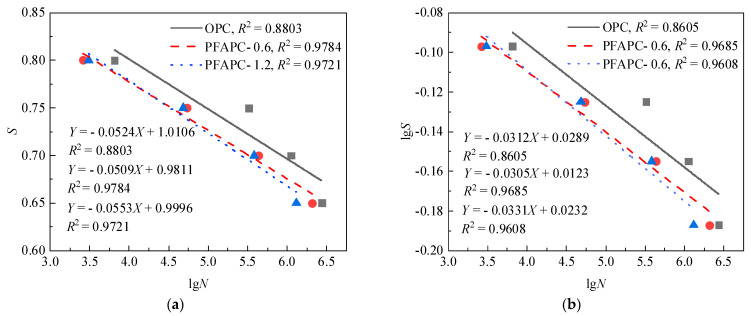
Average life *S–N* curve: (**a**) single-logarithm and (**b**) double-logarithm fatigue equations.

The test findings and the aforementioned boundary requirements can both be satisfied by this ideal form of the fatigue equation. *S–N* curves are traditionally created using the stress level *S* as the ordinate and the average fatigue life as the abscissa [4]. As illustrated in Figure 7, the standard *S–N* curves of OPC and PFAPC were constructed in this study using the average fatigue life in shown Figure 5.

Figure 7 shows that the correlation coefficients of the PFAPC fitting lines were all very high, with the exception of a slightly lower OPC, demonstrating that *S*, lg*S*, and lg*N* each had a solid linear relationship in the PFAPC power function model. According to the fitting correlation, the fitting impact of the single-logarithm form was marginally superior to that of the double-logarithm form. As a result, the following single-log and double-log fatigue equations can be used to calculate the average fatigue life of OPC and PFAPC:

$$\mathrm{OPC}:\left\{ \begin{array}{l} S=1.0106-0.0524\mathrm{lg}N\\ \mathrm{lg}S=0.0289-0.0312\mathrm{lg}N \end{array}\right.$$

$$\mathrm{PFAPC}-0.6:\left\{ \begin{array}{l} S=0.9811-0.0509\mathrm{lg}N\\ \mathrm{lg}S=0.0123-0.0305\mathrm{lg}N \end{array}\right.$$

$$\mathrm{PFAPC}-1.2:\left\{ \begin{array}{l} S=0.9996-0.0553\mathrm{lg}N\\ \mathrm{lg}S=0.0232-0.0331\mathrm{lg}N \end{array}\right.$$

To meet the safety performance requirements under cyclic loading in practical engineering, the *P-S-N* fatigue equations of OPC and PFAPC were established under a given failure probability in the form of single- and double-logarithm fatigue equations. The aim was to establish a direct quantitative relationship between the regression fatigue equation and failure probability *F* (or survival rate *P* = 1 − *F*). The fatigue lives of OPC and PFAPC both followed a two-parameter Weibull distribution, according to the analysis in Section 3.3 and Section 3.4. Equation (10) can be used to compute the fatigue life *N_f_* under various failure probabilities *F*:

(10) $$N_{f}=N_{a}{\left[\ln\left(\frac{1}{1-F}\right)\right]}^{\frac{1}{b}}$$

The coefficients *b* and *N_a_* obtained in Table 6 were substituted into Equation (10), and the fatigue lives *N_f_* (equivalent fatigue life) of OPC and PFAPC were calculated with given failure probability *F*, as shown in Table 8.

The data in Table 8 were regressed using Equations (7) and (9). Table 9 provides a summary of the regression coefficients *A*, *B*, lg*a*, and *b* of the single- and double-log fatigue equations, which correspond to various failure probabilities *F*.

Table 9 demonstrates that, regardless of the shape of the single- or double-logarithm form, the correlation *R*^2^ between OPC and PFAPC rapidly declined as the failure probability increased. In the single-logarithmic form of fitting, the correlation of other groups under different failure probabilities was over 0.90, and the correlation of the PFAPC group was above 0.97, with the exception of a slightly lower correlation in OPC when the failure probability was 0.40–0.50. With the exception of a marginally lower correlation in OPC when the failure probability was 0.20–0.50, the correlations of all other groups in the double-log fitting were over 0.90, whereas those of the PFAPC group were above 0.96. This showed that when the failure probability *F* is considered, the equivalent fatigue life *N_f_* of the PFAPC obeyed the two-parameter Weibull distribution with high accuracy. Additionally, the corresponding linear relationship between the single-log fatigue equation and the double-log fatigue equation was essentially established, with the degree of fit for the single-log fatigue equation being slightly higher than that of the double-log equation. This is in line with the results of the fitting correlations between the average fatigue life corresponding to the single- and double-log fatigue equations.

Failure probability *F* had little impact on regression coefficients *B* and *b*, and can be ignored, similar to the majority of the fatigue test results for fiber-reinforced concrete. The single- and double-logarithm fatigue equations of airport pavement concrete, considering failure probability *F*, can be determined by using the average value of *B* and *b* as the general result, as shown below:

$$\mathrm{OPC}:\left\{ \begin{array}{l} S=A-0.0505\mathrm{lg}N\\ \mathrm{lg}S=\mathrm{lg}a-0.0301\mathrm{lg}N \end{array}\right.$$

$$\mathrm{PFAPC}-0.6:\left\{ \begin{array}{l} S=A-0.049\mathrm{lg}N\\ \mathrm{lg}S=\mathrm{lg}a-0.0294\mathrm{lg}N \end{array}\right.$$

$$\mathrm{PFAPC}-1.2:\left\{ \begin{array}{l} S=A-0.053\mathrm{lg}N\\ \mathrm{lg}S=\mathrm{lg}a-0.0318\mathrm{lg}N \end{array}\right.$$

**Figure 8:**
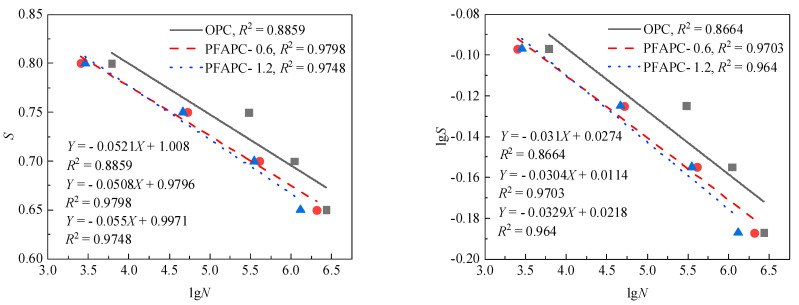
*P-S-N* curve when survival probability *P* = 50% is considered under two-parameter Weibull distribution.

**Figure 9:**
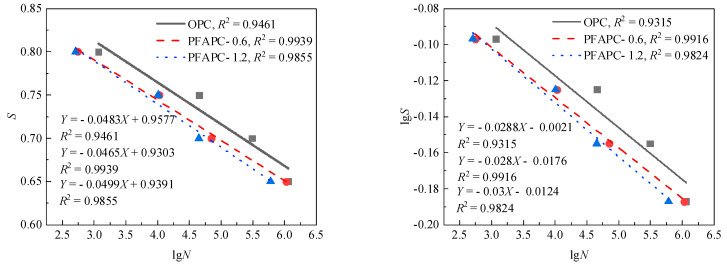
*P-S-N* curve when survival probability *P* = 95% is considered under two-parameter Weibull distribution.

These equations serve as a guide for forecasting the fatigue life of PFAPC with various failure probabilities under various stress levels, when the above formulae are combined with the regression coefficients under various failure probabilities as shown in Table 9. Flexural fatigue testing of concrete generally yields two *S-N* curves with high reference values. The first is the *P-S-N* curve, which corresponds to a survival rate of 50%, or a failure probability of 50%; the maximum fatigue strength of a material can be determined through this curve. The second is the corresponding survival-rate *P-S-N* curve; *P* is 95%, which means that *F* is 0.05 in terms of failure probability. The ultimate strength of the material under conditional fatigue can be determined by this curve, and this value can serve as a general guide for structural design [34,35]. Figure 8 and Figure 9 display the *P-S-N* curves for the two survival rates.

According to a previous study [3], a concrete specimen has an unlimited life if it is not harmed after 2 × 10^6^ cycles of loading. The ultimate fatigue strength of material generally refers to the maximum fatigue stress that the material can withstand under a certain number of cycles, and is usually expressed in the form of static flexural strength percentage in practical application [4,7,24]. Researchers studying the ultimate fatigue strength of concrete materials typically use a cyclic foundation of 2 × 10^6^ fatigue cycles. The OPC and PFAPC ultimate fatigue strength (upper limit of stress level) and conditional ultimate fatigue strength corresponding to a 2 × 10^6^ fatigue life under two survival rates was calculated using Figure 8 and Figure 9, as well as the established single- and double-log fatigue equations, as shown in Table 10.

No difference in the ultimate fatigue strength was estimated by using the single-logarithm and double-logarithm form equations, as shown in the calculation results in Table 10. The ultimate fatigue strengths of OPC, PFAPC-0.6, and PFAPC-1.2 with a survival rate of 50% were found to be 0.68 *f_r_* (where *f_r_* is the static flexural strength of OPC and PFAPC), 0.66 *f_r_*, and 0.65 *f_r_*, respectively, equating to a 2 × 10^6^ fatigue life. The conditional ultimate fatigue strengths of OPC, PFAPC-0.6, and PFAPC-1.2 corresponding to a 2 × 10^6^ fatigue life were calculated as 0.65 *f_r_*, 0.64 *f_r_*, and 0.63 *f_r_*, respectively, when considering a survival rate of 95%. The average fatigue life of the material in the fatigue test was indicated to be larger than 2 × 10^6^ times when the loading stress amplitude was less than or equal to the ultimate fatigue strength, suggesting no fatigue failure. When the survival rate was 50%, the fatigue strength of concrete with POM fibers at 0.6 and 1.2% volume decreased by 2.9 and 4.4%, respectively; when the survival rate was 95%, the fatigue strength fell by 1.5 and 3.1%, respectively. The addition of POM fiber reduced the flexural fatigue performance of airport pavement concrete to a certain extent.

## 4. Conclusions

1. PFAPC specimens exhibit particular ductile failure traits when subjected to fatigue stress, and a three-stage curve can adequately represent the complete fatigue deformation process.
2. POM fiber can somewhat increase the deformation capacity of airport pavement concrete under flexural fatigue loading at different stress levels. The major improvement was observed in PFAPC-1.2. At a high stress level (*S* = 0.80), the maximum midspan deflection of PFAPC-1.2 increased by 37.9%, and the maximum tensile strain increased by 89.2%; at a low stress level (*S* = 0.65), its maximum midspan deflection increased by 101.2%, and the maximum tensile strain increased by 36.5%.
3. PFAPC has a substantially shorter fatigue life than OPC, and starts to decline noticeably at increasing stress levels. At a low stress level (*S* = 0.65), compared with OPC, the fatigue lives of PFAPC-0.6 and PFAPC-1.2 decreased by 23.8% and 52.1%, respectively; at a higher stress level (*S* = 0.75), their fatigue lives decreased by 83.4% and 85.1% respectively. The fatigue life of PFAPC-1.2 was shorter than PFAPC-0.6, except at the 0.80 stress level.
4. The fatigue life of OC and PFAPC well obeyed the two-parameter Weibull distribution, the fitting correlation coefficient *R*^2^ was generally above 0.90, and these conclusions were further verified by K–S testing. The single-logarithm fatigue equation had a marginally higher fit than the double-logarithm fatigue equation, both of which were developed using two-parameter Weibull distributions considering various failure probabilities. The ultimate fatigue strength of airport pavement concrete is marginally decreased by the addition of POM fiber.

## 5. Future Recommended Research

The conclusion shows that POM fibers have different degrees of negative effects on the fatigue life of airport pavement concrete. This is different from the results from other studies of polymer-fiber-reinforced concrete. These findings were obtained under the premise of ensuring the validity of the test results. It is hoped that this conclusion will be taken seriously by more colleagues and more research will be conducted to clarify why these negative effects occur, and how they should be addressed.

## Figures and Tables

**Table 1:** Comparison of fiber properties.

Fiber Type	Density (g/cm^3^)	Strength (MPa)	Elongation Rate (%)	Modulus (GPa)
POM	1.41	1000	15	8.5
Glass	2.7	736	2.45	80
Carbon	1.85	1770	0.1–0.2	180
PP	0.91	285–570	15–25	3.85
Steel	7.85	1100–1300	0.2	200
Basalt	2.65	4500	2.4–3.0	95–115

**Table 2:** Physical and mechanical properties of POM fiber.

Classification	Density (g/cm^3^)	Diameter (mm)	Strength (MPa)	Modulus (GPa)	Elongation Rate (%)	Length (mm)
Straight	1.41	0.2	1000	8.5	15	12

**Table 3:** Mix ratio of airport pavement concrete.

Specimen Type	W/B	Dosage by Volume Fraction (%)	Mix Ratio (kg·m^−3^)					
			Binding Material	Water	Sand	Rough Stone (16~26.5 mm)	Fine Stone (4.75~16 mm)	Water Reducer
OPC	0.37	0.0	383.23	155.97	565.87	886.53	433.84	2.11
PFAPC-0.6	0.37	0.6	383.23	155.97	565.87	886.53	433.84	2.11
PFAPC-1.2	0.37	1.2	383.23	155.97	565.87	886.53	433.84	2.11

**Table 4:** Static mechanical properties of airport pavement concrete.

Specimen Type	Compressive Strength (MPa)		Flexural Strength (MPa)	
	28 d	90 d	28 d	90 d
OPC	54.9	61.5	5.98	6.58
PFAPC-0.6	50.4	67.8	6.06	6.72
PFAPC-1.2	49.6	67.0	5.80	6.45

**Table 5:** Fatigue test results.

Specimen Type	Stress Level (*S)*	Fatigue Life (*N*)	ln*N*	Stress Level (*S*)	Fatigue Life (*N*)	ln*N*	Reliability (*p*)	ln[ln(1/*p*)]
OPC	0.65	1,857,013	14.43	0.70	678,544	13.43	0.833	−1.7
		2,134,386	14.57		810,497	13.61	0.667	−0.904
		2,721,339	14.82		1,049,851	13.86	0.5	−0.367
		3,148,897	14.96		1,126,098	13.93	0.333	0.095
		3,799,215	15.15		1,964,998	14.49	0.167	0.582
	0.75	143,424	11.87	0.80	2874	7.96	0.833	−1.7
		160,161	11.98		3983	8.29	0.667	−0.904
		297,665	12.6		5791	8.66	0.5	−0.367
		495,713	13.11		8816	9.08	0.333	0.095
		526,866	13.17		11,205	9.32	0.167	0.582
PFAPC-0.6	0.65	1,587,895	14.28	0.70	200,820	12.21	0.833	−1.7
		1,783,115	14.39		222,100	12.31	0.667	−0.904
		1,906,017	14.46		498,761	13.12	0.5	−0.367
		2,390,813	14.69		579,841	13.27	0.333	0.095
		2,743,466	14.82		667,508	13.41	0.167	0.582
	0.75	20,921	9.95	0.80	1290	7.16	0.833	−1.7
		45,949	10.74		1556	7.35	0.667	−0.904
		61,921	11.03		2798	7.94	0.5	−0.367
		63,512	11.06		3615	8.19	0.333	0.095
		77,928	11.26		3862	8.26	0.167	0.582
PFAPC-1.2	0.65	899,331	13.71	0.70	148,758	11.91	0.833	−1.7
		1,098,774	13.91		177,894	12.09	0.667	−0.904
		1,367,851	14.13		387,642	12.87	0.5	−0.367
		1,426,593	14.17		591,324	13.29	0.333	0.095
		1,754,092	14.38		622,321	13.34	0.167	0.582
	0.75	20,924	9.95	0.80	1395	7.24	0.833	−1.7
		38,705	10.56		1775	7.48	0.667	−0.904
		41,334	10.63		2509	7.83	0.5	−0.367
		68,429	11.13		4559	8.42	0.333	0.095
		71,754	11.18		5081	8.53	0.167	0.582

**Table 6:** Linear regression analysis results.

Specimen Type	*S*	*b*	*β*	*N_a_*	*R* ^2^
OPC	0.80	1.5765	14.1144	7731	0.9815
	0.75	1.3832	17.8123	391,445	0.9073
	0.70	2.0635	29.0677	1,311,392	0.8811
	0.65	3.0131	45.0098	3,072,493	0.9762
PFAPC-0.6	0.80	1.7136	13.7908	3127	0.9299
	0.75	1.6312	18.0887	65,460	0.8972
	0.70	1.4844	19.5544	526,128	0.8872
	0.65	3.8471	56.3491	2,297,196	0.9279
PFAPC-1.2	0.80	1.5133	12.4142	3653	0.9375
	0.75	1.7214	18.8608	57,337	0.9463
	0.70	1.268	16.5623	470,617	0.9137
	0.65	3.41	48.4032	1,460,807	0.98

**Table 7:** KS test results.

Specimen Type	*S*	*D_i_*	*D_c_*	Result
OPC	0.80	0.166	0.563	Accept
	0.75	0.2213		
	0.70	0.2818		
	0.65	0.1502		
PFAPC-0.6	0.80	0.2379	0.563	Accept
	0.75	0.2648		
	0.70	0.2408		
	0.65	0.2141		
PFAPC-1.2	0.80	0.1925	0.563	Accept
	0.75	0.2296		
	0.70	0.2405		
	0.65	0.1976		

**Table 8:** Equivalent fatigue life calculation results.

Specimen Type	*S*	Fatigue Life *N_f_* at Failure Probability *F*					
		0.05	0.10	0.20	0.30	0.40	0.50
OPC	0.80	1175	1855	2986	4020	5049	6127
	0.75	45,719	76,932	132,350	185,772	240,859	300,327
	0.70	310,893	440,665	633,939	795,715	947,016	1,097,980
	0.65	1,146,517	1,455,906	1,867,654	2,182,214	2,458,506	2,720,592
PFAPC-0.6	0.80	553	841	1303	1713	2113	2525
	0.75	10,597	16,475	26,099	34,794	43,364	52,287
	0.70	71,137	115,530	191,536	262,706	334,628	411,017
	0.65	1,061,442	1,279,843	1,555,506	1,757,185	1,929,157	2,088,444
PFAPC-1.2	0.80	513	826	1356	1848	2344	2867
	0.75	10,211	15,513	23,989	31,502	38,812	46,341
	0.70	45,224	79,783	144,189	208,723	277,076	352,481
	0.65	611,380	755,072	940,940	1,079,679	1,199,616	1,311,940

**Table 9:** Regression coefficients considering failure probability.

Specimen Type	*F*	*A*	*B*	*R* ^2^	lg*a*	*b*	*R* ^2^
OPC	0.05	0.9577	−0.0483	0.9461	−0.0021	−0.0288	0.9315
	0.10	0.9715	−0.0494	0.9328	0.0061	−0.0295	0.9169
	0.20	0.9861	−0.0505	0.9164	0.0146	−0.0301	0.8991
	0.30	0.9952	−0.0512	0.9047	0.0199	−0.0305	0.8864
	0.40	1.0022	−0.0517	0.8948	0.024	−0.0308	0.8759
	0.50	1.008	−0.0521	0.8859	0.0274	−0.031	0.8664
PFAPC-0.6	0.05	0.9303	−0.0465	0.9939	−0.0176	−0.028	0.9916
	0.10	0.9438	−0.0478	0.9934	−0.0096	−0.0287	0.9892
	0.20	0.958	−0.049	0.9903	−0.0012	−0.0294	0.984
	0.30	0.967	−0.0498	0.9869	0.004	−0.0298	0.9793
	0.40	0.9738	−0.0503	0.9834	0.008	−0.0301	0.9748
	0.50	0.9796	−0.0508	0.9798	0.0114	−0.0304	0.9703
PFAPC-1.2	0.05	0.9391	−0.0499	0.9855	−0.0124	−0.03	0.9824
	0.10	0.9552	−0.0515	0.9879	−0.0028	−0.0309	0.9828
	0.20	0.9721	−0.053	0.9864	0.0071	−0.0318	0.9791
	0.30	0.9826	−0.0539	0.9831	0.0133	−0.0323	0.9744
	0.40	0.9905	−0.0545	0.9791	0.0179	−0.0326	0.9694
	0.50	0.9971	−0.055	0.9748	0.0218	−0.0329	0.964

**Table 10:** Ultimate fatigue strength under two-parameter Weibull distribution.

Specimen Type	*P* (%)	Equation Form	Ultimate Fatigue Strength (%*)*
OPC	50	Single logarithm	68
		Double logarithm	68
	95	Single logarithm	65
		Double logarithm	65
PFAPC-0.6	50	Single logarithm	66
		Double logarithm	66
	95	Single logarithm	64
		Double logarithm	64
PFAPC-1.2	50	Single logarithm	65
		Double logarithm	65
	95	Single logarithm	63
		Double logarithm	63

## Data Availability

Not applicable.
